# The PDB and the ribosome

**DOI:** 10.1016/j.jbc.2021.100561

**Published:** 2021-03-18

**Authors:** Peter B. Moore

**Affiliations:** Department of Chemistry, Yale University, New Haven, Connecticut, USA

**Keywords:** PDB, structure, model, ribosome, crystallography, cryo-EM, BMRB, Biological Magnetic Resonance Data Bank, Hma, *Haloarcula marismortui*, LRS, large ribosomal subunit, PDB, Protein Data Bank, SRL, sarcin-ricin loop, SRS, small ribosomal subunit

## Abstract

This essay, which was written to commemorate the 50th anniversary of the Protein Data Bank, opens with some comments about the intentions of the scientists who pressed for its establishment and the nature of services it provides. It includes a brief account of the events that resulted in the determination of the crystal structure of the large ribosomal subunit from *Haloarcula marismortui*. The magnitude of the challenge the first ribosome crystal structures posed for the PDB is commented upon, and in the description of subsequent developments in the ribosome structure field that follows, it is pointed out that cryo-EM has replaced X-ray crystallography as the method of choice for investigating ribosome structure.

## Happy birthday, PDB

The 50th anniversary of the Protein Data Bank (PDB) is an occasion worth celebrating. It has done everything its founders could possibly have hoped for. At the beginning, it was a tiny operation embedded in the Chemistry Department of Brookhaven National Laboratory. Today it is a large, multi-institutional organization (Rutgers, UCSD, and UCSF) that is part of international consortium called the Worldwide PDB, the members of which are the RSCB (USA), PDBe (Europe), PDBj (Japan), and the Biological Magnetic Resonance Data Bank (BMRB).

The PDB would not exist today if a succession of public-spirited scientists had not devoted themselves to it. Walter Hamilton was its first manager, and following his untimely death in 1973, Tom Koetzle took over. Tom was succeeded by Joel Sussman in 1994. In 1999, the PDB was moved from Brookhaven to Rutgers, the home institution of its next director, Helen Berman. Fifteen years later, Helen was succeeded by Stephen Burley. We owe them a lot.

Credit is also due to the managers of the agencies that have supported the PDB financially: primarily DOE, NSF, and NIH. At the time of it was founded, the PDB was a very strange duck indeed, and in the early 1970s, no one would have had any reason to be surprised if these agencies had refused to fund it. Happily, their managers understood the importance of the benefits that might be realized if some of the research funds they were responsible for were used to support an enterprise that was, and still is, a public utility. The rest, as they say, is history.

## What is a “structure”?

As everyone knows, the PDB is a publicly accessible repository of information about the positions of atoms in biological macromolecules, and the essence of what it provides its users are sets of coordinates that specify the locations of most, if not all, of the atoms in particular macromolecules. Even though biochemists routinely use the word “structure” to describe these coordinate sets, it is important to realize that they are not structures. The structure of a macromolecule is the actual arrangement of its constituent atoms in three dimensions, about which our knowledge will always be imperfect to some degree. A coordinate set is an atomic *model* of a structure that rationalizes the data produced by a particular experiment done to characterize it.

The conflation of the word “structure” with coordinate sets was of little concern in the early days of the PDB because they were all arrived at the same way, namely by fitting sequence into density maps that had been obtained by X-ray (or neutron) crystallography. It is difficult to fit sequence into maps that have resolutions much worse than 3 Å, and it usually requires prior knowledge of both a macromolecule’s sequence and the structures of its monomers. (The latter derive from monomer crystal structures that have been solved at resolutions far higher than most macromolecular crystals afford.) The end product is a list of the coordinates and B-factors of a large fraction of the atoms in the macromolecule of concern. Crystallographic coordinates are estimates of the average positions of the atoms in the asymmetric unit of a crystal, and while, ideally, B-factors should report only on the variations in their positions caused by thermal fluctuations, but static crystal disorder, experimental error, and model error contribute to them too. Finally, for most crystallographic models, the number of independent reflections measured to obtain the electron (or scattering length) density map on which it is based exceeds the number of coordinates specified by the model, and if you are willing to concede the validity of the monomer bond length, and bond angle restraints used to refine crystal structures, they are massively overdetermined. This being the case, the practice of using the word “structure” to describe sets of atomic coordinates, *i.e.*, models, was largely harmless.

In the last decade or so, the PDB has begun hosting an ever-increasing number of molecular models based on cryo-EM data. They too are produced by fitting sequence into three-dimensional maps, and if the resolution of an EM map is reasonably high, *i.e.*, better than, say, 4 Å, the number of parameters specified by these molecular model will be smaller than the number of terms in the Fourier transform of the parent map out to its resolution limit. (There are some subtleties here that we will ignore.) Thus, although there are still unresolved issues surrounding the interpretation of cryo-EM density, and the validation of EM models, it is reasonable to think of models derived from high-resolution EM maps as being equivalent to crystallographic models.

The models of proteins derived from NMR data, which began to appear in the PDB in the mid-1980s, differ qualitatively from their crystallographic and EM cousins. In the first place, the number of experimental restraints in the data sets used to arrive at NMR models is often significantly less than the number of atomic coordinates they specify, and many of them are not all that restraining. Consequently, the data are usually compatible with a family of related models, five or ten of which may be published so that users can decide for themselves how well determined they are by the data. Fortunately, it is almost invariably the case that when a structure is determined by both NMR and crystallography, the resulting models are obviously alternative renderings of the same structure.

Far more problematic are the increasing number of models in the PDB based on data that are much sparser, *e.g.*, models based on EM maps having resolutions too low to permit the fitting of sequence into density *ab initio*. These models are often generated by inserting atomic resolution structural models into lower-resolution maps, and then, sometimes, massaging them to improve the correspondence between models and maps. This is an advanced form of an activity sometimes referred to as “blobology.” It is worth preserving these models in a public database so that others can work with them, but it would probably be wise to keep them in a database separate from the PDB so that users will not confuse them with models that far more adequately supported experimentally.

The reason it is important to make such distinctions is that, viewed using a molecular graphics program, every structural model that specifies atomic positions looks as authoritative as every other, and this can lead to problems. For example, at a poster session many years ago, two scientists, who had been mining information about nucleic acid conformation from the nucleic acid structures then in the PDB, told me that hydrogen bond distances in base pairs are much better determined by NMR than they are by crystallography. They were dismayed when I replied that the data NMR spectroscopists collect from nucleic acids is largely silent about base pair geometries, and consequently, *faute de mieux*, they often assume that the hydrogen-bonding geometries of all the base pairs they identify are canonical. Lest the reader think that I am bad-mouthing my NMR colleagues - who are all salt of the earth, I hasten to add—there is nothing peculiar to NMR about this. For example, if you were to compile statistics about the Cα–Cβ distance in all the leucine residues in all the X-ray models of proteins in the PDB, you would doubtlessly find that they hardly vary at all, the reason being that bond distances like these are usually tightly restrained during refinement. This story has two morals: (a) you should not draw conclusions from models the provenance of which you do not understand, and (b) models are not the same as structures. Nevertheless, consistent with common practice, models will often be referred to as structures below.

Even the structures in the PDB that have been determined by what some might describe as “gold standard methods,” *i.e.*, crystallography and EM, are not all created equal. Low-resolution structures are never as accurate as high-resolution structures, and in many cases, little can be done about it because the resolutions of structures are often limited by the properties of the crystals or of the EM specimens used to determine them. That said, similarity in resolution is no guarantee of similarity in quality because the thoroughness with which data have been analyzed and models refined can make a difference too. For example, it is perfectly possible these days for a laboratory whose members know little about crystallography to solve a crystal structure, and submit a set of coordinates to the PDB. No one should be surprised if the accuracy of such a coordinate set might turn out to be lower than it would have been if the same structure had been solved by a group experienced in the art. (Given the amazing speed with which EM is evolving into a user-friendly technique, problems of this sort may soon arise in that domain also.) It also needs to be pointed out that some of the structures in the PDB are less than they should be because the data were manipulated in ways they should not have been, *e.g.*, see (1).

Several years ago, I suggested to Helen Berman that the PDB ought to optimize the structures it curates before releasing them to the public, which in the case of crystal structures would mean rerefining them all at the very least. She was doubtlessly horrified by this cheeky proposal, but in a friendly tone of voice she conceded that many of the structures in the PDB were not all that they could or should be, but went on to point out that the PDB is an archive of published models, and for that reason, responsibility for model quality rests with depositors rather than the PDB. What the PDB does do that is helpful in this regard is run diagnostic test to assess the quality of coordinate sets, and it includes the results of those tests in the files it distributes to the public so that users can judge how well models have been refined. As depositors know, the diagnostic information the PDB generates during the deposition process often leads them to refine their structures further, which is unquestionably a Good Thing. The PDB might be wise to include a test that looks at the B-factors in the crystallographic coordinate sets. Erratic variations in atomic B-factors are a sure sign that a crystal structure has problems.

It is much harder to assess the quality of EM structures than it is to do the same for X-ray crystal structures. The physics of the diffraction of X-rays by crystals is very well understood (*e.g.*, (2)), and it is a simple matter to compute the intensities of the reflections that some crystal would produce if its structure corresponded exactly to the model proposed for it. Thus the validity of a crystallographic model can be assessed by comparing computed intensities with measured intensities (but see (3)). Nothing so simple is possible for EM structures because EM maps display the way the electrostatic potential varies within macromolecules. Consequently, EM maps are strongly affected by atomic charges, both full and partial, *e.g.*, (4). Thus, in principle, in addition to specifying the identities, positions, and temperature factors of all the atoms in a molecule, an EM model should also specify their charges, which they currently do not. It is not obvious how to extract this information from EM maps. Nor is it clear how you would compute maps from structural models even if you could. Thus, the microscopists still have some work to do, but given the youth of their technique, and the rate at which it is advancing, there is every reason to be optimistic that these problems will be addressed.

## On the state of play in 1971

From the point of view of most readers of this article, the PDB has been around forever. However, I am ancient enough to remember what life was like before it existed, and I know a little bit about how it came to be because of the conversations I had at the time with Frederic Richards, a senior colleague who was a prominent member of the group that lobbied for its establishment.

Those interested in what the field of structural biology was like at the time the PDB was founded can do no better than consult the volume that emerged from the 1971 Cold Spring Harbor Laboratory Symposium on Quantitative Biology (5). The title of that meeting was “Structure and Function of Proteins at the Three-dimensional Level,” and an amazing group of scientists participated. Nine of them had already won, or would go on to win, the Nobel Prize. Crystallographers dominated the proceedings, but there were also contributions from NMR spectroscopists, and electron microscopists, as well as from the end users of structures, *e.g.*, enzymologists. It was obvious that the rate at which structures were being solved was about to increase dramatically both because the number of laboratories doing macromolecular crystallography was growing and because technical advances had already dramatically reduced the amount of labor it took to solve protein crystal structures. The PDB was set up to deal with three of the problems created by these happy developments: publication, public access, and preservation.

## What do you mean “publish”?

As everyone knows, biochemists announce their findings in “papers,” and for the benefit of the young, I point out that the reason they are called papers is that at the time the PDB was founded, they were invariably documents printed on actual pieces of paper, hard though that may be to believe. In that era, papers were accessed by going to facilities called libraries, which were places where books and journals were stored—a nostalgic tear comes to the eye.

Prior to the publication of the first macromolecular crystal structures, biochemical papers tended to be straightforward. They might include a description of a purification, provide a molecular weight or two, or some Michaelis parameters, or in cases of extreme complexity, an amino acid composition, or even an amino acid sequence. That information, plus the essence of the conclusions the authors had drawn from their experiments, could usually be conveyed to the reader on half a printed page. In that same era, papers describing small-molecule crystal structures were not much different. They usually included a table giving the coordinates and temperature factors of all the atoms in some molecule, of which might be 30, or 40, plus a few drawings that could be relied on to give the reader a good idea of what that molecule of concern looked like.

Nothing so informative is/was even remotely possible for papers describing macromolecular crystal structures. In the first place, publishers were never going to print tables listing the coordinates and temperature factors of all the nonhydrogen atoms in a protein. A table of this sort could easily contain a thousand entries, and what was anyone going to do with a printed list that huge? Furthermore, the illustrations in these papers were often problematic, as they still are. It is hard to make illustrations of macromolecular structures that are really informative. Moreover, using the information these papers provided, it was impossible for readers to satisfy themselves that the structures they described had been solved properly, or that the conclusions drawn by the authors from their structures were sound. Thus, despite the best intentions of their authors, macromolecular structure papers often amounted to little more than victory announcements.

If you really cared about a published structure, you could ask the group responsible to send you the coordinates. If you were lucky, a box of IBM cards or a magnetic tape might eventually appear in the (snail) mail. As for converting those coordinates into something to look at, you were on your own. The only other option was to collaborate with that group, a gambit that might or might not work out, depending on personalities and priorities. Thus, by comparison with other kinds of biochemical papers, only a tiny fraction of what had been learned was revealed to the public when a crystallographic group published a protein structure.

Richards knew this, and in an effort to do better, he and his colleague, Harold Wyckoff, published a spiral-bound book that described the crystal structure they had obtained of RNase S in far greater detail than would ever have been possible in even a long series of conventional papers (6). It included a lot of red-green stereo illustrations, which were state of the art at the time, and it was the first volume in a series entitled, “Atlas of Molecular Structures in Biology,” of which David Phillips and Fred Richards were the editors. Fred and David hoped that every group that solved a protein structure would eventually contribute a volume to the Atlas. However, as far as I know, only one other such volume ever appeared (7). The failure of this initiative doubtlessly reflected a reluctance on the part of crystallographic groups to invest the time required to write books of the quality Fred and David were looking for.

Now that the PDB has come of age, no one would think of embarking on a publishing venture of that kind. Today, anyone who has a laptop or desktop computer can examine any of the published macromolecular structures he or she might want, in however much detail he or she might wish, in the comfort of his or her own office. You do not even have to go to the library! Furthermore, those who really care about a crystal structure can recover the electron density map from which it derives and can test its validity by computing (F_o_ − F_c_) difference maps, for example. The PDB, of course, is where people get the information needed to do these things, and thanks to the internet, it is delivered instantly; no need to wait for the mail. Thus, once a structure has been released by the PDB, it has been well and truly published. Furthermore, the release-on-publication policy, on which sponsoring agencies and journals now insist, guarantees public access to structures almost as soon as they have been determined. It no longer depends on where you work or who you know.

## Preserving knowledge

Preservation was the other big issue on Richards’ mind. Few of us work on the same problem for an entire career; our interests change, and we move on. Consequently, data we value highly today is unlikely to seem all that important 20 years from now, and the older data become, the less likely they can be recovered from the archives, let alone be made sense of. This problem presents itself in its starkest form when the individual responsible ascends to the Great Laboratory in the Sky. Fred understood this, and he realized that every structure represents a substantial investment of human labor and public funds. Unless steps were taken to prevent it, coordinate sets, *i.e.*, structures, were bound to get lost, and in his view, this was unconscionable.

My colleague Thomas Steitz used to argue that the best repository of information about the structure of a crystal is the crystal itself. If you know how to grow crystals of some protein, which the methods section of the paper in which its structure was announced often reveals, the data you collect from the crystals of that protein you prepare for yourself are likely to be at least as good as the data used to solve its structure to begin with, and thus you may emerge with a better understanding of that protein’s structure than the people who first solved it. This approach to coordinate preservation may make sense for those who run crystallographic laboratories, as Tom did, but it is not a viable option for anyone else. It is better for the community if coordinates are preserved at the time structures are published, and better yet, if the data from which coordinates were derived are also archived so that structures can be re-evaluated later, should the need arise.

For those who have produced structures, the archiving function of the PDB provides a benefit that its founders may not have anticipated. Over the course of the determination of a structure, the people involved are likely to have collected many data sets and to have refined it repeatedly. Their notebooks may refer to scores of files containing data sets and to hundreds more containing the results of refinements. After they leave, it can be a penance for the remaining members of their group to identify the particular refinement and data set that gave rise to the published version of that structure, and retrieval of the relevant files may also be problematic. However, if the final product got deposited in the PDB, which is mandatory these days, both the structure and the associated data set will be forever available to the members of the group responsible for it, no matter what has happened since. No need for data forensics.

I note in passing the fragility of the electronic archiving systems used to store data today, including the ones that support the PDB and the online scientific journals on which we all rely. Databases disappear if they are not maintained. In addition, the physical stabilities of the memory devices on which databases depend leave a lot to be desired, and technological change can also be destabilizing. For example, outside the Smithsonian Institution, who still has a magnetic tape deck, let alone a reader for IBM cards? Will the information now stored in the PDB still be available in 2121? We should all be concerned about this.

## On the science the PDB has fostered

Of the myriad ways that have been found to use the data in the PDB, three stand out. First, and most obviously, there is nothing more empowering for a biochemist than knowledge of the structures of the macromolecules involved in some process he or she cares about. At the time the structure of a macromolecule is first solved, it invariably explains most of what had been learned about it previously by other means. (When this is not so, alarm bells should go off because it is a sure sign that something odd is going on.) Structures are also the *sine qua non* for both the design and interpretation of experiments based on techniques such as site-directed mutagenesis, fluorescence labeling, etc. Thus, the access to structures the PDB provides is invaluable to biological scientists of all stripes.

Second, the PDB contains most of what is known today about the three-dimensional structures of proteins and nucleic acids and hence about the relationship between sequence and structure. It is also a precious resource for those interested in the relationship between structure and function because the many structures in the PDB are those of functionally significant macromolecular complexes and of enzymes with substrates, substrate analogs, and inhibitors bound. Consequently, the PDB is essential for everyone whose research depends on extracting general truths from structures. I note in passing that Fred Richards was one of the first to use the PDB this way. In the mid-1970s, he became interested in characterizing the way atoms are packed in the interiors of proteins, and the methods he devised for describing the surfaces and interiors of proteins are still widely used (8, 9).

Third, the PDB has become enabling for those who solve crystal structures. In the early days of protein crystallography, diffraction patterns had to be phased experimentally. At first, this was done by multiple isomorphous replacement, but later on anomalous scatter techniques also came into use. However, by the late 1960s, it had been realized that the structures of macromolecular crystals can also be solved computationally, using a technique called molecular replacement, which bypasses experimental phase determination altogether (10, 11). Molecular replacement exploits the fact that usefully accurate, first approximation models of macromolecules of unknown structure can be obtained by assuming that their structures are the same as those of macromolecules of known structure that have related sequences. The larger the number of structures in the PDB, of course, the higher the probability that the PDB will include such a structure, and the more powerful molecular replacement becomes.

## The genesis of the first ribosome crystal structures

Ribosomes are the ribonucleoprotein particles found in the cytoplasm of almost all cells that catalyze messenger RNA-directed protein synthesis. Their substrates are aminoacyl tRNAs, and their products are proteins. They were discovered in the 1950s, and by the late 1960s, at a broad-brush level anyway, their mechanism of action was well understood (see (12)). For example, by 1970 it was clear that ribosomes are composed of two subunits, the larger one, *i.e.*, the large ribosomal subunit (LRS), being about twice the size of the smaller one, *i.e.*, the small ribosomal subunit (SRS), and that the SRS brokers the interactions between mRNAs and aminoacyl tRNAs that ensure that the genetic code is correctly translated, while the LRS includes the active site that catalyzes the formation of peptide bonds (for recent reviews see (13, 14)).

From the outset, if was obvious that important insights into the mechanism of gene expression would emerge if atomic resolution crystal structures could be obtained of ribosomes, but although there were earlier indications that ribosomes can crystallize (15), it was not until 1980 that the first three-dimensional crystals of ribosomes were grown that were large enough to work with (16).

It can take a long time to solve the structures of crystals. For example, hemoglobin crystals were first observed in the early decades of the 19th century, long before the discovery of X-rays, let alone X-ray crystallography (17), but it was not until 1968 that the first atomic resolution crystal structure of hemoglobin was published (18). By comparison, the time that elapsed between the first report of ribosome crystals and the first atomic resolution ribosome structures was next to nothing, 1980 to 2000 (16, 19, 20, 21). However, by 1980, macromolecular crystallography was a mature discipline, and so, in fact, it is reasonable to ask why it took so long.

Ribosome crystals diffract weakly because their unit cells are large, and at room temperature, their diffraction patterns rapidly fade away due to radiation damage. Since in addition the first crystals were also not all that well ordered, the resolutions of the diffraction patterns obtained from them were disappointingly low. Nevertheless, crystallization studies continued in both Berlin and, later, Pushchino, *e.g.*, (22). Ultimately, not only were better ordered ribosome crystals obtained, *e.g.*, (23), but by the early 1990s, the radiation damage problem was under control too. It had been demonstrated that high-quality data sets can be collected from frozen ribosome crystals using X-ray area detectors and the bright X-ray beams produced by synchrotron light sources (24).

At that point, the only problem that remained was finding a way to phase ribosome diffraction patterns, and it was not obvious how to go about it. On the one hand, molecular replacement was out of the question because there were no structures in the PDB that were even remotely related to those of intact ribosomes or ribosomal subunits. On the other hand, the techniques available for experimental phasing also seemed problematic. They all depend on adding heavy atoms to specific sites in macromolecular crystals in ways that do not perturb their structures and then measuring the resulting changes in diffraction intensities. If the molecular weight of the asymmetric unit in the crystals of concern is large, as it is for all ribosome crystals, a large number of high-Z atoms will have to be added per asymmetric unit to make those intensity changes big enough to measure accurately. The good news is that the larger the molecular weight of the asymmetric unit, the larger the number of binding sites it is likely to contain for any given heavy atom compound. The bad news is that if the number of sites is too large, it may be impossible to determine their locations from the changes in intensities they cause (see below), and if that cannot be done, no phase information will be obtained.

By the mid-1990s, four groups had taken up the phasing challenge. Due to actions taken subsequently by the Nobel Foundation, the names of the leaders of three of them are familiar to most: Ada Yonath (Weizmann), Venki Ramakrishnan (Laboratory of Molecular Biology), and Thomas Steitz (Yale). The fourth group was led by Harry Noller (University of California, Santa Cruz). Had the crystals of 70S ribosomes the Santa Cruz group worked on diffracted to atomic resolution, the Nobel Foundation might have had a hard time deciding what to do in 2009.

Venki recently published a book that tells the story of how he and his group solved the structure of the SRS from *Thermus thermophilus* (Tth), and it includes a lot of information about what his competitors did (25). However, we do not know how they saw it. Neither Ada nor Harry has (yet) written anything similar, and, sadly, Tom never will. He passed away in the fall of 2018. However, I can comment on the approach the Yale group took, because my group collaborated with Tom’s on solving the crystal structure of the LRS from *Haloarcula marismortui* (Hma).

Tom and I had talked about ribosome crystallography many times over the years. It interested me because I had been working on the ribosome for most of my career, and I had studied its three-dimensional organization for many years by neutron scattering in solution (26), a technique related to crystallography. Since the explanatory power of the information we had obtained by neutron scattering was limited, to put it kindly, I was eager to see if something better could be done. Tom was interested because the ribosome is the largest of the macromolecules involved in gene expression, and Tom’s goal in life was to determine the crystal structures of as many of them as possible. For both of us, the objective was to explain the functional properties of the ribosome in structural terms.

Nothing came of these conversations until ∼1994, when we agreed that if Tom was to find a postdoc willing to take on the ribosome, my group would at the very least provide whatever biochemical support he or she might need. Not long thereafter Tom persuaded Nenad Ban to become that postdoc, and he moved to Yale in the fall of 1995. Nenad’s project was not for the faint of heart. On the one hand, there was no guarantee that anyone would ever succeed in phasing ribosome diffraction patterns; failure was definitely an option. On the other hand, if it could be done, it was entirely possible that someone else might do it first.

In 1995, shortly before Nenad moved to Yale, a conference on ribosomes was held in Canada at Victoria, BC, and I attended, as I had attended almost all of its predecessors. There I learned that the phase problem was no closer to being solved than it ever had been; Nenad was in no immediate danger of being scooped. Even more important, the pictures presented there of the three-dimensional model of the *Escherichia coli* ribosome that Joachim Frank and his colleagues had recently obtained from cryo-EM images demonstrated that the image reconstruction approach to structure determination had just taken a giant step forward. I returned to New Haven convinced that ribosome diffraction patterns could be phased by molecular replacement using EM models of ribosomes of the quality Joachim was now able to produce.

Two important decisions were made as Nenad got down to work later that year. First, rather than investing the time it would have taken to develop a new kind of ribosome crystal of our own, we would reproduce the ones that had been reported to diffract the best, which were the crystals of the LRS from Hma that Ada had first prepared a decade earlier (23). Second, we would pursue molecular replacement using cryo-EM ribosome models as the test objects. To that end we formalized the collaboration I had discussed with Joachim at the Victoria meeting, and soon thereafter, we sent him some Hma LRS so that he and his colleagues could produce the cryo-EM model required.

We knew that the resolution of the model we hoped Joachim would produce was unlikely to exceed ∼15 Å, and hence the resolution limit of the phases obtained when it was used for molecular replacement would be no better. However, we also realized that a set of phase estimates even that limited in resolution would probably have a huge impact on our ability to extract phase information from heavy atom isomorphous replacement experiments. They would enable us to use difference Fourier maps rather than difference Patterson maps to locate the sites where heavy atoms had bound in our crystals. The advantage of the former over the latter is easy to understand. If the number of heavy atoms bound per unit cell is *N*, the number of peaks in the corresponding heavy atom difference Fourier map will also be *N*, and the position of each peak in the map will be that of a heavy atom-binding site. By contrast, the number of peaks in the corresponding difference Patterson map, which is computed from measured intensity changes assuming that the phases of all reflections are zero, will be *N* (*N* − 1), and their positions will be determined by the lengths and directions of all of the heavy atom to heavy atom vectors in the unit cell, rather than by the positions of those heavy atoms, *per se*. If N is greater than a few dozen, say, peak overlap alone can make it impossible to extract positional information from heavy atom difference Patterson maps.

Not content to rely only on molecular replacement, Nenad also began reaching out to inorganic chemists around the world to see if they would give us samples of the heavy atom cluster compounds they had synthesized. The reason heavy atom cluster compounds were interesting is that, at low resolution, a molecule that contains *N* atoms of atomic number *Z* will diffract X-rays about the same way a single “super” atom would that contains *NZ* electrons. Thus, appreciable changes in the intensities of reflections may be observed if only a few of these compounds bind per asymmetric unit, and if the number bound is small, it will probably be possible to determine their locations using difference Patterson methods. The problem with cluster compounds is that the amplitude of the contribution a cluster compound makes to diffraction patterns falls very fast as resolution increases, and in the limit of high resolution, it is no greater than would be seen if N atoms of the metal of concern had bound to the unit cell independently. Thus, the cluster compound approach to phasing, like the EM-based molecular replacement approach, was likely to work only at low resolution.

This “low resolution first” approach to phasing had implications for our data collection strategy. Until we managed to phase our diffraction patterns at low resolution, it was not going to be necessary to collect data out to the resolution limit of the crystals we were working with; lower-resolution data sets would do. As it happened, satisfactory data sets of this sort could be collected from Hma LRS crystals using the bending magnet beam lines at the National Synchrotron Light Source (NSLS) that were run by Bob Sweet and his colleagues for the benefit of macromolecular crystallographers. By 1995, interest in those beam lines had begun to wane not only because some of the other NSLS beam lines Bob’s team managed had been retrofitted with insertion devices that made them far more capable, but also because there were better beam lines at other light sources. Consequently, not only was it easy to schedule all the time we wanted on Bob’s bending magnet beam lines, he would sometimes offer us extra time when gaps arose unexpectedly in the schedule. We could easily take advantage of these offers because it only takes 3 h to get from Yale to Brookhaven National Laboratory, where NSLS was located. (“Was” is appropriate here because NSLS was decommissioned several years ago. *Sic transit gloria mundi*.)

As luck would have it—and it is always good to have luck on your side—one of the first heavy atom cluster compounds Nenad tested, which contained 18 tungsten atoms, binds with high affinity to only a single site in the asymmetric unit of the orthorhombic, C222_1_ crystals of the LRS he was working with, the position of which he could easily determine by difference Patterson methods. However, about the time this highly encouraging result was obtained, we began having trouble reproducing these crystals. What we began getting instead were crystals that had almost the same unit cell dimensions and angles, diffraction patterns that had nearly the same symmetry, and Bragg reflection intensities similar to those of the C222_1_ crystals. However, the W18 difference Patterson maps obtained from the two types of crystals were not the same, which was baffling.

At about this juncture, Joachim and his colleagues, Pawel Penczek and Robert Grassucci, sent us the model they had obtained for the Hma LRS. A plausible way of packing their model in the unit cell of the C222_1_ crystals was soon found that was also consistent with intensities of the low-resolution reflections obtained from them. The difference Fourier maps calculated for the one-site derivative discussed above using X-ray intensities and EM-derived phases revealed the presence of a single, high-occupancy site in the asymmetric unit of the C222_1_crystals, the location of which was identical to the one that had been found for it using Patterson methods. At that point, we knew the ribosome phasing problem had been solved.

Using Frank’s model, it was also possible to determine the way subunits are packed in the second type of crystal, which turned out to be monoclinic, P2_1_ crystals that were merohedrally twinned. The twinning explained why the symmetry of the diffraction patterns produced by these crystals was about the same as that of the diffraction patterns produced by the C222_1_ crystals, which would not have been otherwise. The only difference between the two crystal forms turned out to be a small displacement in a single subunit–subunit contact. Once this problem had been sorted out, data obtained from P2_1_ crystals in which the twin fraction was low could be used to improve our phase estimates for the diffraction patterns obtained from C222_1_ crystals. Later, Jeff Hansen discovered that the packing at this critical contact depends on the concentration of monovalent salt in the solutions used to stabilize Hma LRS crystals. If the concentration of salt in these solutions was kept at saturating levels C222_1_ symmetry would be preserved, but if the concentration was reduced by as little as 5%, C222_1_ crystals would morph into twinned P2_1_ crystals with no obvious signs of distress.

Tom added a second postdoc to the team in 1997, Poul Nissen, and by early 1998, Nenad and Poul had produced a 9 Å resolution electron density map of the LRS from Hma that had been phased by multiple isomorphous replacement and anomalous scattering methods using three heavy atom derivatives. That map, the approach used to obtain phases, and the conclusions drawn from it were reported in a paper published in 1998 (27). Technically, it is the most important of the long series of papers we published on the Hma LRS because it demonstrated that structures can be obtained from ribosome crystals.

Once we had gotten this far, low-resolution, bending magnet data sets were no longer enough, and we began using the insertion device beamlines at NSLS to obtain data sets that extended to limit of the resolution afforded by our crystals. Later on, we also began using Andrzej Joachimiak’s beamline at the Advance Photon Source (Argonne National Laboratory), which was even more powerful. A year later, a 5 Å resolution electron density map of the LRS emerged from these higher-resolution data sets (28). (NB: One of the heavy atom derivatives used to obtain that map was generated by soaking osmium hexamine into Hma LRS crystals. It binds to 38 sites in the Hma LRS, and consequently that derivative would have been useless if low-resolution phases had not already been available.) The 5 Å resolution map was frustrating. While it was obvious from that map that the Hma LRS consists of both RNA and protein—no surprise there—it was impossible to build sequence into the density with any certainty. What you could do instead was identify regions that correspond to ribosomal components of previously known structure and fit those structures into them. (In the last decade, this approach to the interpretation of less than atomic resolution maps has become standard operating procedure in cryo-EM world.)

From my point of view, the most interesting of these fitting exercises involved a ∼25-nucleotide stem loop called the sarcin-ricin loop (SRL) that is found in all 23S rRNAs. Its structure was already known, and a program that searches for features in electron density maps that have shapes resembling a known structure was used to find that particular needle in the 23S rRNA haystack. To our surprise, it identified two of them: one on the subunit interface surface of the LRS, about where we expected the SRL to be on the basis of a plethora of other evidence, and the other at a location that made no sense. After the structure was solved, we discovered that instead of being a stem loop, that second feature was a place where three strands of RNA come together, and that the structure in the middle of that junction is almost identical to that of the bulge in the middle of the stem of the SRL. The program had told us the truth.

**Figure 1:**
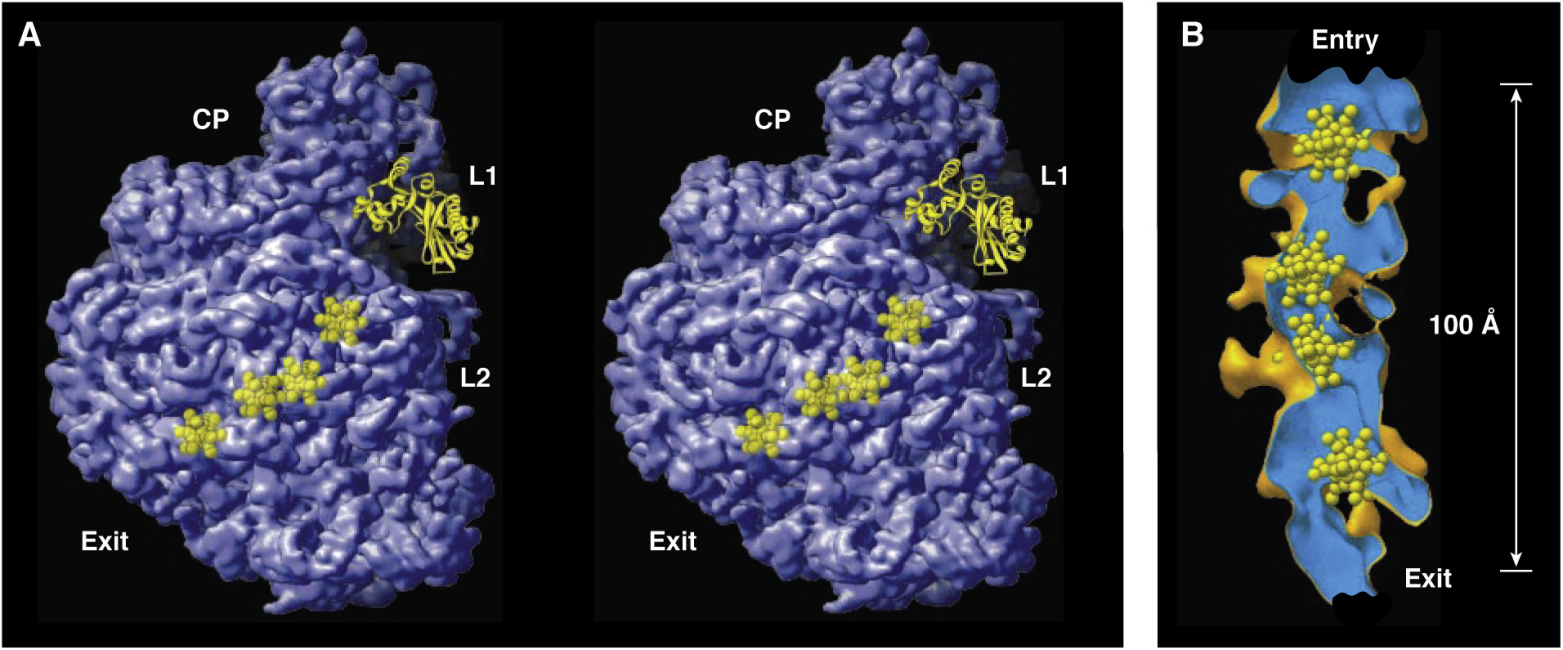
**The binding sites of a W**_**11**_**cluster compound in the exit tunnel of the Hma LRS.***A*, a stereo pair of the large ribosomal subunit with the part proximal to the viewer cut away to reveal the four sites where the W_11_ compound binds. *B*, a close-up of the interior of the exit tunnel showing the wall of its lumen and the four W_11_ sites. Reproduced from (28) with the permission of the publisher.

The exit tunnel of the LRS, through which nascent peptides pass as they are synthesized, was clearly evident in the 5 Å resolution electron density map, and proof that molecules of substantial size can diffuse into and out of its lumen emerged when the positions where heavy atom cluster compounds had bound to the LRS were superimposed on this map. One of those compounds was a molecule containing 11 tungsten atoms, and four of the sites where it binds to the Hma LRS are *inside* the exit tunnel (Fig. 1).

In the summer of 1999, a few weeks before our 5 Å paper was published, the successor to the 1995 ribosome meeting was held in Denmark at Helsingør. There presentations were made by all of the groups engaged in ribosome crystallography. Tom spoke for the Yale contingent (29), and the substance of his talk was similar to that of the 5 Å paper (28). Venki discussed the progress his group had made with the SRS from Tth, for which he had a 5.5 Å resolution map (30). His talk came as a total surprise to most of the audience because it was the first time he had said anything in public about his whole-ribosome crystallographic work. Ada’s presentation revealed that she and her colleagues had given up on the Hma LRS, possibly because of the space group instabilities that had given us so much trouble, and that they had decided to concentrate instead on the Tth SRS for which they had a 7 Å resolution map (31). Harry’s talk dealt both with the results of some chemical probing experiments his group had done, and with the work they were doing with crystals of the 70S ribosome from Tth, for which they had a 7.8 Å resolution map (32). Thus, by the end of the 1999 meeting, it was clear to all that atomic resolution structures for ribosomes and ribosomal subunits were imminent.

The response of the ribosome community to this epoch-making development was mixed. For decades, many of its members had been trying to elucidate the structure of the ribosome using a wide variety of noncrystallographic methods, and they were depressed by the realization that the results they had worked so long and so hard to obtain would shortly become irrelevant. In the summary talk I gave at the end of the meeting, I did what I could to cheer them up (33). I pointed out that once structures become available, they would be able to use their techniques to do far more penetrating experiments on the ribosome than they had ever been able to carry out before, which turned out to be true, but I am not sure they were all that comforted.

**Figure 2:**
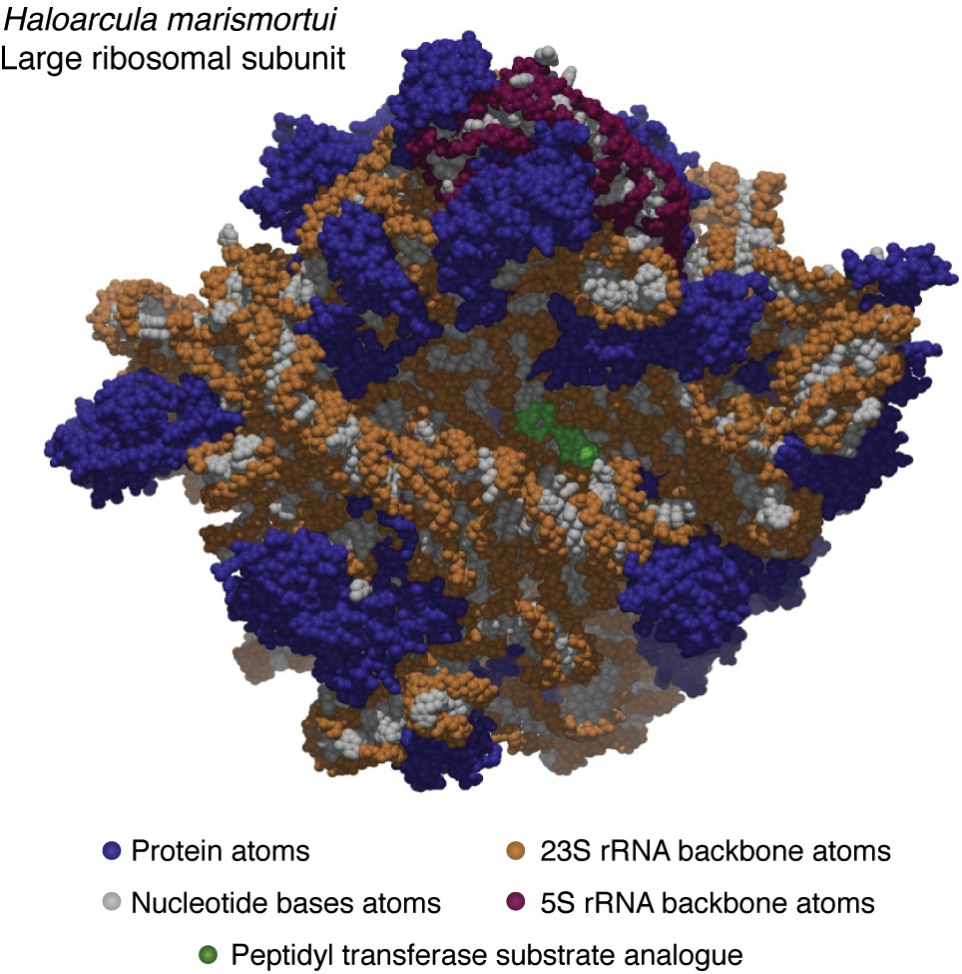
**A space-filling model of the subunit interface surface of the Hma LRS.** Nonhydrogen atoms are shown as van der Waals spheres. Protein atoms are *blue*. Atoms belonging to nucleotide bases are *gray*. Backbone atoms belonging to 23S rRNA are *brown*, and those associated with 5S rRNA are *purple*. The atoms of a peptidyl transferase substrate analogue bound in peptidyl transferase center are *green*. This image was prepared for the author by Professor Poul Nissen.

Just a few months after the 1999 meeting, we obtained the first electron density map of the Hma LRS that had a resolution high enough so that sequences could be fit into it unambiguously, and I remember feeling overwhelmed by the magnitude of the task that now confronted us. It was all hands on deck. The postdocs were so desperate for help that I was allowed to fit 23S rRNA sequence into the part of the map that corresponds to domain 1. Sequence was built into the protein parts of the map by beginning graduate students. Despite the large number of people involved, it took months to get the job done. While it was in progress, some new crystals were taken to APS for data collection, one of which diffracted to a resolution much higher than that of any we had examined before: 2.4 Å. On Aug. 11, 2000, the Yale group published a structure of the LRS from Hma at that resolution, and at somewhat lower resolution, a structure of the same subunit with a substrate analog of the peptidyl transferase reaction bound (Fig. 2) (19, 34). Three weeks later, Ada and her coworkers published a structure of the Tth SRS that they had derived from a 3.3 Å resolution electron density map (20), and 1 month after that, Venki’s group published a much more accurately interpreted version of the same structure that was based on a 3 Å resolution electron density map (21).

**Figure 3:**
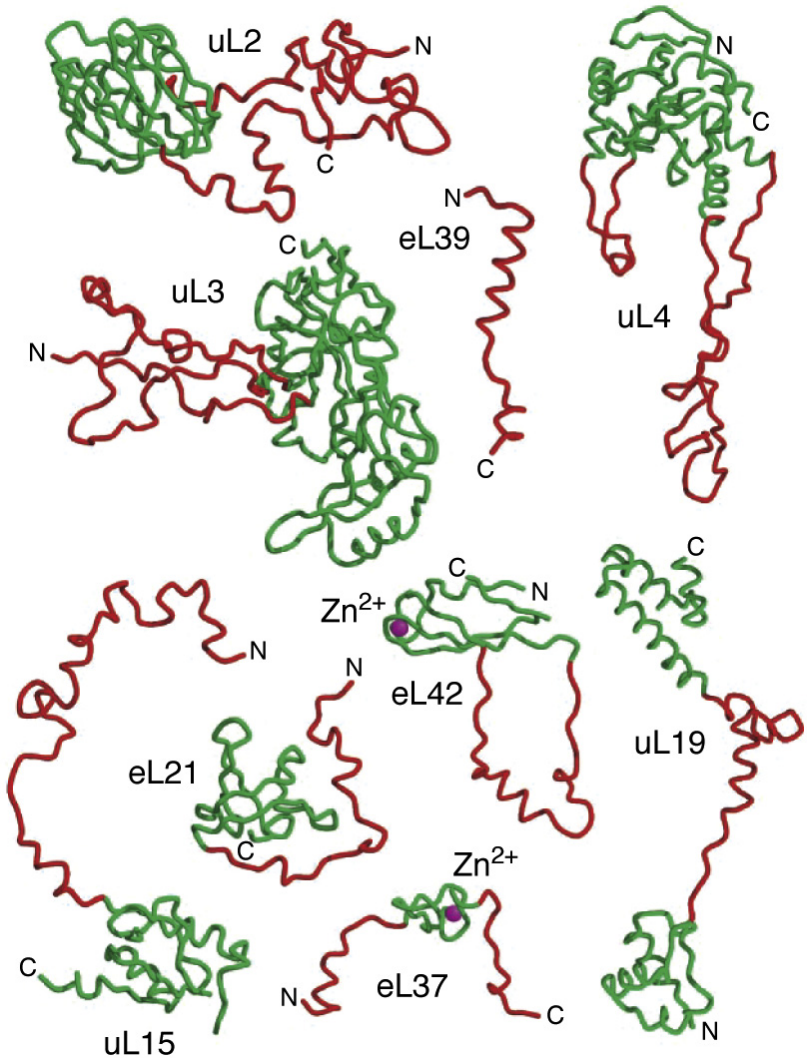
**Some proteins in the Hma LRS that have tails.** Globular domains are *green*. Tails are *red*. *Purple spheres* are Zn^2+^ ions. Proteins are named following the rules provided in (52). (Note: Protein eL42 is called L44e in many publications) (Redrawn from (19)).

We can do no more here than highlight a few of the most interesting findings that emerged from these structures. In the first place, they revealed that the models for the secondary structures of the three prokaryotic rRNAs that had been generated by sequence comparisons were spot on. Their first versions had appeared many years before (35, 36, 37). Second, the structure obtained of the Hma LRS with a peptidyl transferase substrate analog bound demonstrated that the site that catalyzes peptide bond formation during protein synthesis is made of RNA (34). It also became clear that nucleotides in 16S rRNA play a critical role in ensuring the fidelity with which mRNA sequences are translated into protein sequences (38). Thus, there could no longer be any doubt that, at heart, the ribosome is a ribozyme. Third, the conformations of many ribosomal proteins turned out to be far more eccentric than anyone had imagined. Many consist of a globular domain that has a long tail extending from it, the conformation of which is dictated by its interactions with rRNA, and a few are nothing but tail. Figure 3 shows some examples. The globular domains of those ribosomal proteins that have them, which most do, tend to be found on the surfaces of both subunits, and the tails usually extend into their RNA-rich interiors. Because of the way protein tails permeate the interior of the LRS, the fraction of the material that is protein in the center of that particle is about the same as it is on its exterior surface. Fourth, and most important of all, these structures permanently altered the way people think about and do experiments on the ribosome, which is what atomic resolution structures of macromolecules have been doing for biochemists since the first one was published in 1960 (39).

## The ribosome and the PDB

So it was that at the end of the summer of 2000, the PDB had to confront the leading edge of what soon became an avalanche of ribosome structures. Ribosome depositions were challenging for the PDB because ribosomes are huge. The molecular weight of the typical bacterial ribosome is ∼2.5 × 10^6^, and it consists of three RNA molecules and 50 to 60 proteins of ordinary molecular weight. To make matters worse, ribosomes have no internal symmetry, which means that the molecular weight of the asymmetric unit of a ribosome crystal is an integer multiple of the molecular weight of an entire ribosome, or, possibly, merely, a ribosomal subunit. Thus, the amount of structure described in a single ribosome deposition can easily be equivalent to that of 50 to 100 ordinary enzymes, and the accompanying diffraction data sets are correspondingly large. It is a credit to the PDB that it survived this onslaught.

By 2000, it had become standard practice to require not only that all structures be deposited in the PDB prior to publication, but also that they be released to the public upon publication. I remember feeling that this was unfair. We had risked a lot working on this problem, and our structure represented a major investment of time and resources, including many man-months spent fitting structure into electron density maps. Why shouldn’t we be granted a few “extra” months to harvest the fruits of our labor? (I doubt I am the only one who has ever felt this way.) Nevertheless, the relevant grant managers and journal editors were adamant; no release, no publication. In the end, I think they were right, and, in fact, we suffered little harm as a result because others were reluctant to engage with our structure for fear that by the time they discovered anything worthwhile, we would have beaten them to it.

If the crystal structure of a macromolecule is known, it is trivial to solve the structures of crystals of the complexes it forms with small molecules. Many small molecules bind to the ribosome, including ∼100 antibiotics that inhibit bacterial ribosomes. Thus, the three founding ribosome structures were swiftly followed by scores of structures that showed how these ribosomes and ribosomal subunits interact with small molecules. From the point of view of the PDB, each of these follow-on depositions was just as massive as the first ones had been; there was to be no rest for the weary.

The founding ribosome structures, which derived from two prokaryotic organisms, *H. marismortui* and *T. thermophilus*, were quickly followed by ribosome structures from two other prokaryotic species: *Deinococus radiodurans* (40) and *E. coli* (41). Both were solved by molecular replacement using the appropriate “founding” ribosome structures as the test objects. No one was in the slightest surprised that molecular replacement had sufficed to solve these structures. RNA sequence comparisons had long since provided compelling evidence that the structures of prokaryotic ribosomes are all closely similar (42), an inference strongly supported by comparisons of ribosomal protein sequences. It should be added in passing that if a structure can be solved by molecular replacement, the amount of time it will take to interpret maps, *i.e.*, to fit sequence into electron density, will be much shorter than it would have been if it had been solved using experimental phases because the test model will guide the interpretation of the density.

Surprisingly, these founding ribosome structures also proved useful as molecular replacement models for crystals of eukaryotic ribosomes. Cytoplasmic ribosomes from eukaryotes are almost twice as large as prokaryotic ribosomes, and while the RNA components of these two classes of ribosomes are homologous, eukaryotic rRNAs are substantially larger than prokaryotic rRNAs, and prokaryotic ribosomes lack homologs for many eukaryotic ribosomal proteins. Thus, it was not obvious that the phases obtained for the diffraction patterns of crystals of eukaryotic ribosomes by molecular replacement using models based on prokaryotic structures would be accurate enough to be useful, but they were. Molecular replacement contributed to the determination of the crystal structure of both the *Saccharomyces cerevisiae* 80S ribosome (43) and the 60S ribosomal subunit from *Tetrahymena thermophila* (44).

## Recent developments in the structural biology of the ribosome

With the appearance of the first eukaryotic crystal structures, the golden age of ribosome crystallography drew to a close. Over the last decade, cryo-electron microscopy has emerged as the method of choice for determining the structures of large macromolecular complexes like the ribosome. Compared with crystallography, its advantages are its capacity to cope with inhomogeneous preparations, the tiny amounts of material it consumes, and of course, the fact that crystallization is not required. That said, structure determination by cryo-EM is not as straightforward as it is sometimes made to sound. Specimen preparation can be difficult because of the tendency of macromolecules to interact in unfortunate ways with grid surfaces and/or air-water interfaces. However, satisfactory methods for preparing ribosome specimens for cryo-EM were found long before the resolution revolution took place (45), and cryo-EM has since yielded a large number of ribosome structures we might still be waiting for if crystallography was the only tool at our disposal. Among them are structures for many of the intermediates in the protein synthesis cycle that are hard to prepare, *e.g.*, (46), for ribosome assembly intermediates, *e.g.*, (47), and for mitochondrial, *e.g.*, (48, 49), and chloroplast ribosomes, *e.g.*, (50).

A decade ago, I thought that the excitement that had been generated in 2000 by the publication of the first ribosome crystal structures would subside once structures had been obtained for roughly a dozen key intermediates in the prokaryotic protein synthesis cycle (13), and it might have done but for the advent of high-resolution cryo-EM. For example, the synthesis of ribosomal components and their assembly is an enormously important process in all cells, and in eukaryotes, it is almost rococo in its complexity. There is an enormous amount still to be learned about it, and cryo-EM is the ideal tool for investigating its structural aspects. On an entirely different front, cryo-EM has taught us not only that mitochondrial ribosomes are incredibly different from the cytoplasmic ribosomes of both eukaryotes and prokaryotes, which we already knew from biochemical investigations, but also that they vary wildly in structure from one species to the next (51). What does protein synthesis look like when it is catalyzed by these odd-ball particles? Again, cryo-EM is likely to play an important part in addressing these problems. Thus, the structural biology of ribosomes remains a viable area of scientific inquiry that likely produces its share of surprising results in the decades to come, and as it continues to evolve, the PDB is certain to play an important role in helping it advance.

## Conflict of interest

The authors declare that they have no conflicts of interest with the contents of this article.
